# Social Media Use, Body Shape Concern, and Self-Esteem Among Saudi Student Nurses: A Cross-Sectional Descriptive Study

**DOI:** 10.3390/bs16071074

**Published:** 2026-07-01

**Authors:** Ferdinand Gonzales, Reem Humaidi Alalawi, Sumathi Robert Shanmugam, Regie Buenafe Tumala, Lorraine Estadilla, Liezl Pangilinan, Abdulrahman Sulaim Almutairi, Eric Anies, Jupiter Cajigal, Sahar Hamdi El Sayed Abdelmaksoud, Sahar Mahmoud Abdulla Hashim, Allen Joshua Dominguez, Kawther Eltayeb Ahmed

**Affiliations:** 1 Department of Medical-Surgical Nursing, College of Nursing, King Khalid University, Abha 61421, Saudi Arabia; rhalalwy@kku.edu.sa (R.H.A.);; 2 Department of Maternity and Pediatric Nursing, College of Nursing, Princess Nourah bint Abdulrahman University, Riyadh 11671, Saudi Arabia; srshanmugam@pnu.edu.sa; 3 Medical-Surgical Department, College of Nursing, King Saud University, Riyadh 12372, Saudi Arabia; rtumala@ksu.edu.sa; 4 Monash Health, Clayton Victoria, Melbourne 3168, Australia; 5 College of Applied Medical Sciences, Shaqra University, Shaqra 11961, Saudi Arabia; a-almutairi@su.edu.sa (A.S.A.);; 6 Department of Nursing, College of Applied Medical Sciences, University of Bisha, Bisha City 67611, Saudi Arabia

**Keywords:** social media, body image, self-concept, students, nursing, Saudi Arabia, cross-sectional studies

## Abstract

**Introduction:** This cross-sectional study examined associations between Social Media Use (SMU), Body Shape Concern (BSC), and Self-Esteem (SE) among Saudi student nurses. We assessed demographic differences in these variables and tested whether SE moderated the SMU–BSC relationship. **Methods:** We conducted a cross-sectional correlational study with 324 student nurses recruited through convenience sampling at King Khalid University. Data were collected via Google Form between January and February 2025 using the SMU Questionnaire, BSQ-8A, and Rosenberg Self-Esteem Scale. **Results:** Higher SMU and greater BSC were each associated with lower self-esteem (ρ = −0.334 and −0.492, *p* < 0.001, shared variance = 11.2% and 24.2%). SMU and BSC were positively correlated (ρ = 0.393, *p* < 0.001). Female students reported marginally higher SMU and BSC than males, but effect sizes were negligible (Cohen’s d = 0.07 and 0.02). Second-year students scored highest across all three variables, with small effect sizes (η^2^ = 0.02–0.03). SE did **not** moderate the SMU–BSC relationship (interaction term: *p* = 0.113), though both SMU and SE independently predicted BSC. **Conclusions:** Among Saudi student nurses, higher social media use and greater body shape concern were associated with lower self-esteem. Female and second-year students showed marginally higher scores, though effects were small. Contrary to the vulnerability-stress hypothesis, self-esteem did not moderate the social media–body concern relationship. Interventions may need to combine media literacy with self-esteem enhancement rather than relying on self-esteem approaches alone.

## 1. Introduction

The use of social media has drawn attention to its association with body image and self-esteem (SE), particularly for at-risk groups such as student nurses. It plays an important role in the academic and personal lives of student nurses (Fardouly & Vartanian, 2016). Social Media (SM) refers to applications enabling people to create, share, or exchange information and interact with content including texts, images, audio, videos, and user-generated material using mobile and internet technologies (Kotler et al., 2022). These platforms offer youths opportunities for self-expression and interaction with larger audiences, contributing to identity formation over time (Meier & Johnson, 2022). Platforms such as Instagram, Facebook, and TikTok affect the interplay of online identity customization and mental health consequences, particularly self-image and body dissatisfaction (Rehman, 2025; Masri-Zada et al., 2025). Students often experience negative Body Shape Concern (BSC) due to increased social comparison and exposure to unrealistic social media content during intensive training (Abdulwahab et al., 2024; Jiotsa et al., 2021; Miljeteig & von Soest, 2022). In this study, BSC refers specifically to dissatisfaction with body size and shape, representing one dimension of the broader construct of body image (Heilat et al., 2026; Cash, 2004). In this study, we use this term throughout to maintain consistency with our measurement instrument.

Social media use has shown an association with BSC and lower SE. Body Image Dissatisfaction is more prevalent among social media users because it increases when comparing themselves to “ideal” images posted online (Zeeni et al., 2018; Marengo et al., 2018). Young women tend to develop SE problems due to the amplification of beauty and thin images on social media, creating unrealistic beauty standards (Yang et al., 2020; Tylka & Sabik, 2010). The association between social media and unhealthy eating behaviors and related mental health implications, including anxiety and depression, is well documented (Xiong, 2023; Chung et al., 2020). Previous research has shown that SMU is associated with body image problems similar to those faced by other demographics (Zaw & Azenal, 2021). However, very little research examines nursing students specifically—individuals who work long hours physically and emotionally while using social media. Existing research has focused on broader topics such as mental health overall and competency with social media, rather than body image and SE related to SMU (Sun et al., 2023).

The role of SE as a moderator in the relationship between BSC and SMU has yet to be studied among nursing students. Several studies suggest that low SE is associated with negative body image thoughts and interactions with social media (Valkenburg et al., 2017). This gap is important given the increased stress and anxiety commonly reported within nursing education environments, which may be associated with increased susceptibility to social media (Zaw & Azenal, 2021). Furthermore, existing literature attempts to analyze body image through the gender lens, which is too simplistic and fails to capture the complexity involved. Male nursing students likely face considerable challenges and sociocultural pressures regarding their body image, yet they seem to be neglected in research. This points towards a need for more heterogeneous approaches focused on nursing students’ diversity (Sun et al., 2023). This research aims to address this lack of focus through examination of an under-analyzed population. It examines how specific vulnerabilities arise from difficult-to-manage academic, clinical, and professional training. Student nurses face emotional and physical challenges, particularly from overexposure to glorified images on social media. Hence, the study focuses on social media-related issues involving body image and SE among students from diverse educational backgrounds during this challenging phase. Filling this void is likely to improve mental health support in nursing programs, enhancing the development of future healthcare providers both personally and professionally. The current study uses SE as a moderator rather than a mediator for both theoretical and methodological purposes. From a theoretical standpoint, moderation theory determines whether the strength or direction of the association between Social Media Usage (SMU) and Body Dissatisfaction differs at varying levels of SE. This perspective fits within the vulnerability–stress model. Individual characteristics, such as low SE, can either be associated with or attenuate the effects of environmental stressors, such as exposure to social media usage, on outcomes (Rosenthal & Tobin, 2023).

Mediation would assess the role of SE as an explanatory variable, examining the mechanisms by which SMU is related to body dissatisfaction. This addresses a different theoretical process. Due to the cross-sectional nature of this study, mediation is less appropriate than moderation because mediation typically relies upon longitudinal or experimental designs to establish temporal precedence (Maxwell & Cole, 2007).

Therefore, this study seeks to answer three research questions: (1) What are the associations between SMU, BSC and SE among student nurses? (2) Do student nurses exhibit statistically significant differences in SMU, BSC and SE based on demographic factors? and (3) Does SE serve as a moderator between SMU and BSC among student nurses? Within this model, SMU serves as the independent variable, BSC as the dependent variable, and SE as the proposed moderating variable. All relationships found in this study could be viewed as only an association based on a cross-sectional research design. The terms “independent” and “dependent” variables are used as a heuristic for the analytical model and do not indicate which variable preceded the other temporally.

## 2. Methods

### 2.1. Design

This research was designed to be a cross-sectional-correlational study to ascertain the relationship of SMU, body image, and SE among student nurses.

### 2.2. Participants/Setting

The subjects in this research are the student nurses of King Khalid University from the three different branches. It has at least 1200 students enrolled in the first-year nursing until internship programs. Using the OpenEpi website (http://www.openepi.com/Menu/OE_Menu.htm; accessed on 26 December 2024), the researchers determined the sample size needed. First, the researchers entered the “total population” as 1200 into OpenEpi, then selected an anticipated “proportion or response distribution” of 50% (0.5), a “margin of error (precision)” of 5% (0.05), and a “confidence interval” of 95%. The finite population correction factor was applied. Based on these parameters, the calculated sample size was 291. Anticipating approximately 10% non-response, the target sample was set at 324. Due to time constraints and access issues, convenience sampling was employed as the recruitment strategy to obtain willing participants.

Post hoc power analysis was conducted using G*Power 3.1. For the moderation analysis, assuming a small-to-medium interaction effect (f^2^ = 0.05), α = 0.05, and a sample of 324, the achieved power was 0.89, exceeding the conventional threshold of 0.80. For correlation analysis, with N = 324 and α = 0.05 (two-tailed), the minimum detectable correlation was ρ = 0.11 with 80% power, and ρ = 0.14 with 95% power

### 2.3. Data Collection

Following approval by King Khalid University’s Institutional Review Board, the researchers contacted the key person to facilitate collaboration. These key persons helped in determining which students met the study’s requirements. The key persons gave the researchers contact details so they could make their connections. After that, the researchers forwarded the link to the Google Form. Information regarding the study and their rights as participants can be found at the link. Following the information, informed consent was obtained, which included instructions that said they would be giving their consent if they answered the questionnaire. Data collection took place between January and February 2025. The two-year interval between initial IRB approval (March 2023) and data collection was due to administrative delays in securing departmental collaboration and coordinating with student representatives across the three university branches.

### 2.4. Questionnaire

There were three questionnaires used in this study. The SMU questionnaire, Body Shape Questionnaire (BSQ-8A), and Rosenberg Self-Esteem Scale (RSE):

The SMU Questionnaire, developed by Xanidis and Brignell (2016), is a 9-item tool designed to capture social media usage and its problematic aspects, using a five-point Likert scale from 0 (Never) to 4 (Always), with higher scores indicating greater usage and issues as well as higher emotional dependence.

The Body Shape Questionnaire-8A (BSQ-8A), an abbreviated version of the original 34-item BSQ (Bash et al., 1993), was used to assess BSC and dissatisfaction. The BSQ-8A comprises 8 items rated on a 6-point Likert scale from 1 (Never) to 6 (Always), with higher total scores indicating greater BSC and dissatisfaction. The BSQ-8A has demonstrated good psychometric properties in previous research (Bash et al., 1993)

The Rosenberg Self-Esteem Scale is a 10-item measure of global self-worth. Items are rated on a 4-point Likert scale from 1 (Strongly Disagree) to 4 (Strongly Agree), with 5 reverse-scored items. Total scores range from 10 to 40, with higher scores indicating higher SE (Rosenberg, 1965).

Meanwhile, Body mass index (BMI) was calculated from self-reported height and weight data collected via the questionnaire. While self-reported BMI may introduce slight underestimation bias (Connor Gorber et al., 2007), it is a widely accepted approach in large survey research.

These questionnaires were subjected to validation by the four experts in the field (three research postgraduates and one supervisor nurse researcher). These four experts unanimously agreed that items were valid to address the research objectives/the intended constructs/the study’s variables/the key concepts being investigated. Moreover, 15 students participated in a pre-test to make sure the questionnaires were reliable. Strong internal consistency was seen in the results for every instrument. In particular, the Body Shape Questionnaire (BSQ-8A) had a Cronbach’s alpha of 0.89, the Rosenberg Self-Esteem Scale (RSE) had a Cronbach’s alpha of 0.91, and the social media questionnaire had a Cronbach’s alpha of 0.88. Excellent reliability is indicated by these high alpha values, which imply that each questionnaire’s items consistently measured the desired constructs.

The questionnaires were originally developed in English. For this Arabic-speaking sample, all instruments were translated into Arabic using a forward–backward translation procedure. Two bilingual translators independently translated the instruments into Arabic, and a third bilingual expert back-translated them into English to ensure conceptual equivalence. Discrepancies were resolved through discussion among the research team. A panel of four nursing and psychology experts reviewed the Arabic versions for cultural appropriateness and clarity prior to the pilot test.

To further examine the structural validity of the Arabic translations, exploratory factor analysis (EFA) was conducted for each instrument using principal axis factoring (PAF) with Promax oblique rotation. Factor retention was determined by parallel analysis (Horn, 1965), the Kaiser criterion (eigenvalue > 1), and examination of the scree plot. For the SMU Questionnaire, parallel analysis and the Kaiser criterion both supported a one-factor solution (eigenvalue = 4.94, explaining 54.8% of variance; Cronbach’s α = 0.88). The BSQ-8A also showed a clear unidimensional structure (eigenvalue = 4.93, 61.6% variance; α = 0.89). The RSE demonstrated a dominant general factor (eigenvalue = 5.19, 51.9% variance; α = 0.91) with a secondary method factor reflecting item wording direction (positive vs. negative), consistent with previous research (Marsh, 1996; Tomas & Oliver, 1999). Model fit indices for one-factor solutions were acceptable across all three instruments (RMSEA < 0.06, CFI > 0.95, SRMR < 0.08; see Table 1). These results support the structural validity of the Arabic versions within the current sample, though confirmatory factor analysis in independent Saudi samples remains warranted.

### 2.5. Ethical Consideration

The Institutional Review Board (IRB) at King Khalid University approved this study (protocol number KKU-2023-007) on 20 March 2023. The approval was valid for a period of two years and was renewed in January 2025 prior to data collection. All participants’ rights, confidentiality, privacy, and anonymity were fully protected throughout the study.

### 2.6. Statistical Analysis

Statistical analyses were performed utilizing SPSS software, version 26 (IBM Corporation, Armonk, NY, USA). The statistical approach was structured into four steps:

Step One: For categorical variables, descriptive statistics included frequency counts and percentiles. For continuous variables, descriptive statistics consisted of means and their corresponding standard deviations (SDs). To determine whether data met the assumption of normality, the Shapiro–Wilk test was employed. Non-parametric methods were utilized when applicable, as all data sets violated this assumption.

Step Two: Using continuous variables (body mass index [BMI], age, meal frequency, Rosenberg Self-Esteem Scale, Body Shape Questionnaire, and Social Media Use Questionnaire), Spearman rank-order correlation coefficients (ρ) were calculated to evaluate the strength and direction of relationships. Conventional benchmarks were applied to interpret the correlation coefficients: 0.10–0.29 = small, 0.30–0.49 = moderate, 0.50–0.69 = large, and ≥0.70 = very large (Marengo et al., 2018). The proportion of shared variance was evaluated through squared correlations (ρ^2^).

Step Three: Mean differences in social media use (SMU), body shape concern (BSC), and self-esteem (SE) were compared across demographic groups (gender, year level, household income, internet access, area of residence) using Welch’s ANOVA. This method was selected because it is robust to both heteroscedasticity and moderate non-normality, unlike classic ANOVA (Liu, 2015). The magnitude of group differences was assessed by calculating eta-squared (η^2^) values and applying (Cohen, 1988) for pairwise comparisons. Benchmarks for η^2^ were: 0.01 = small, 0.06 = medium, and 0.14 = large (Cohen, 1988); benchmarks for Cohen’s d were: <0.20 = negligible, 0.21–0.60 = small, 0.61–1.20 = medium, and >1.20 = large (Cohen, 1988). Post hoc pairwise comparisons were conducted using Games–Howell tests, which do not require equal variances.

Step Four: Moderated multiple regression was conducted to analyze whether SE moderated the association between SMU (independent variable) and BSC (dependent variable). The model specification was: BSC = β_0_ + β_1_(SMU) + β_2_(SE) + β_3_(SMU × SE) + ε where β_3_ represented the interaction (moderating) effect. Prior to computing the interaction term, both independent predictors were mean-centered to minimize multicollinearity and enhance interpretability (Aiken & West, 1991). OLS regression was used to compute the model parameters. Despite non-normality, OLS regression was appropriate because: (a) the sample size (N = 324) exceeds thresholds where the Central Limit Theorem supports robustness of parameter estimates; (b) regression is generally robust to moderate non-normality in large samples; and (c) mean-centering reduces multicollinearity. Following estimation, several assumptions were examined, including residual plots, the Durbin–Watson statistic to ensure independence of residuals, and variance inflation factor (VIF) to detect multicollinearity. Residual diagnostics confirmed no severe violations.

Significant interaction effects (*p* < 0.05) indicated that the slopes of SMU on BSC differed across levels of SE. Standardized regression coefficients (β) and semi-partial correlations (sr^2^) were computed as estimates of effect size; specifically, semi-partial correlations represent the amount of variance uniquely contributed by each predictor.

## 3. Results

Table 2 presents the socio-demographics of the participants. The findings show a higher representation of female students (60.80%), in second year (33.33%), who had less than 10,000 Saudi Arabian Riyals (SAR) household income. Most participants had internet access at home (94.75%), lived in urban areas (83.33%), and had internet access outside the home (87.65%), and most participants used WhatsApp (88.58%). Lastly, 48.77% reported exercising for 30 min or more daily.

Also in Table 2, it compares the means for the five groups in terms of socio-demographic characteristics, use of social media, concerns about body shape, and SE. As presented in the table, gender showed significant differences for both SMU (*p* = 0.016) and BSC (*p* = 0.034). The female group’s mean SMU score (20.3) was larger than that of the male group (19.7); similarly, the female group showed a higher mean BSC score (17.7) than the male group (17.5). Year level showed significant differences in SMU (*p* = 0.023), BSC (*p* = 0.037), and SE (*p* = 0.050). Second-year students had the largest mean scores across all three measures (SMU: 21.0; BSC: 18.3; SE: 23.7), whereas third-year students produced the smallest mean scores (SMU: 18.8; BSC: 16.5; SE: 22.8). Conversely, neither household income, internet access availability at home, nor area of residence were significant predictors of either SMU or BSC or SE since their respective *p*-values were each greater than 0.05. For non-significant demographic comparisons please see Appendix A.

Correlational analysis (Table 3) revealed significant correlations between studied variables. Moderate positive correlation was found for the relationship between SMU and number of meals per day (ρ = 0.392; *p* < 0.0001) and for the relationship between SMU and BSC (ρ = 0.393; *p*< 0.0001) as well as a negative correlation with SE (ρ = −0.334; *p* < 0.0001), which suggests that greater SMU is associated both with lower SE and more frequent meals. In addition, a weak positive correlation was observed for the relationship between SMU and BMI (ρ = 0.154; *p* = 0.0005). Also, BSC was positively associated with both meal frequency (ρ = 0.373; *p* < 0.0001) and BMI (ρ = 0.506; *p* < 0.0001). These associations suggest that individuals who exhibit greater BSCs eat more frequently and have higher BMI. Additionally, a small positive correlation was observed between BSC and age (ρ = 0.173; *p* = 0.002). Furthermore, a moderate-to-strong negative correlation was observed for the relationship between BSC and SE (ρ = −0.492; ρ^2^ = 0.242), which indicates that there is considerable overlap (approximately 24%) between BSC and SE. This finding supports theoretical models that propose reciprocal relationships; however, further longitudinal research is required to determine whether or not the relationship between BSC and SE is bidirectional. The 95% confidence intervals for these correlations were: SMU–BSC [0.289–0.491], SMU–SE [−0.43, −0.23], and BSC–SE [−0.58, −0.40], indicating precise estimation given the sample size. Finally, it was demonstrated in this study that SE has a negative correlation with both the number of meals consumed per day (ρ = −0.24; *p* < 0.0001) and BMI (ρ = −0.189; *p* < 0.0001), indicating that higher meal frequency and higher BMI were associated with lower SE.

**Figure 1:**
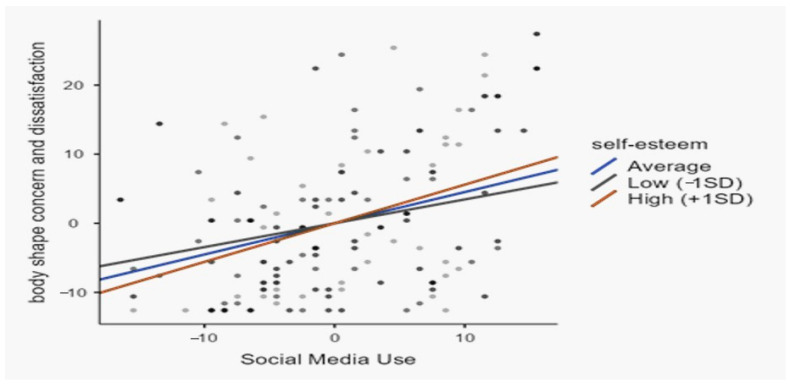
Simple slopes plot of the relationship between SMU and BSC at low (−, 1 SD), mean, and high (+1 SD) levels of SE. The predictor (SMU) and moderator (SE) variables were mean-centered prior to analysis to facilitate interpretation of the interaction term and reduce multicollinearity between main effects and the interaction term (Aiken & West, 1991).

Table 4 and Figure 1 presents the estimates of the moderation model examining whether SE moderates the relationship between SMU (predictor) and BSC. The direct effect of SMU on BSC was significant and positive (Estimate = 0.452, SE = 0.063, t = 7.13, *p* < 0.001), indicating that higher SMU was associated with greater BSC. Similarly, SE had a significant negative direct effect on BSC (Estimate = −0.695, SE = 0.099, t = −7.02, *p* < 0.001), suggesting that higher SE was associated with lower BSC. However, the interaction term (SMU × SE) was not statistically significant (Estimate = 0.022, SE = 0.014, t = 1.59, *p* = 0.113), indicating that SE does not significantly moderate the relationship between SMU and BSC. Therefore, while both SMU and SE independently influence BSC, the effect of SMU on BSC does not significantly change depending on an individual’s level of SE.

## 4. Discussion

### 4.1. Key Findings

This study examined associations between social media use (SMU), body shape concern (BSC), and self-esteem (SE) among Saudi student nurses. It also examined demographic differences and tested the potential moderating role of SE. Three main findings emerged.

First, female student nurses reported significantly higher SMU and BSC than males. However, effect sizes were negligible (Cohen’s d = 0.07 and 0.02), indicating minimal practical significance despite statistical detection (Cumming, 2014). Second, second-year students showed the highest mean scores across all three variables, though effects were small (η^2^ = 0.02–0.03). Third, SMU and BSC were each negatively associated with SE (ρ = −0.334 and −0.492, respectively). SMU and BSC were positively correlated (ρ = 0.393). However, SE did not significantly moderate the SMU–BSC relationship (*p* = 0.113), indicating that the strength of this association did not differ across self-esteem levels.

### 4.2. Gender and Year Level Differences

The statistically significant but negligible gender differences in SMU and BSC partially echo prior studies reporting gender disparities in body dissatisfaction among adolescents (Calzo et al., 2012). However, the current findings suggest a narrower gap among nursing students than in younger or more general populations. This may reflect the homogenization of social expectations within professional training environments, where both male and female students face similar pressures related to appearance and professional presentation (Palomino-Suárez & Aparicio García, 2025). The absence of gender differences in SE further supports this interpretation, suggesting that nursing education contexts may attenuate typical gender-based SE disparities observed in broader populations.

Second-year students scored highest across all three measures. This pattern matches prior research showing that psychological wellbeing fluctuates across academic years (Ghezelbash et al., 2015). This may reflect the transition from foundational coursework to intensive clinical exposure, accompanied by increased social comparison and professional identity formation. However, the small effect sizes indicate that year level explains limited variance, and individual factors likely exert stronger influence. The non-significant effects of household income, internet access, and residence area suggest that intrinsic psychological and educational factors, rather than socioeconomic or infrastructural variables, are more salient predictors of SMU, BSC, and SE in this population.

### 4.3. Correlational Patterns and Their Interpretation

The moderate positive association between SMU and BSC (shared variance = 15.4%) is consistent with prior research linking social media engagement to body dissatisfaction. Mechanisms include social comparison and self-objectification (Papageorgiou et al., 2022; Çınaroğlu & Yılmazer, 2025; Aparicio-Martínez et al., 2019). However, this cross-sectional association is theoretically bidirectional. SMU may be associated with BSC through exposure to idealized images. Alternatively, individuals with pre-existing BSC may seek appearance-focused content. Unmeasured confounders, such as perfectionism and neuroticism, may also be associated with both variables. These correlations represent concurrent associations only and should not be interpreted as causal.

The moderate negative association between BSC and SE (shared variance = 24.2%) indicates substantial overlap. This is consistent with theoretical models suggesting a reciprocal relationship between body image and self-worth. Narrative reviews support this view (Merino et al., 2024) and highlight how body dissatisfaction and societal beauty standards consistently contribute to diminished self-esteem and psychological distress. The weaker but significant SMU–SE association (shared variance = 11.2%) aligns with literature documenting that frequent social media use is correlated with lower psychological wellbeing. Potential mechanisms include upward social comparison and exposure to unrealistic standards (Irmer & Schmiedek, 2023; Mesce et al., 2022). The correlations with BMI and meal frequency further contextualize these relationships. BSC showed a stronger association with BMI (ρ = 0.506) than with SMU (ρ = 0.393). This suggests that actual body characteristics may exert greater influence on body dissatisfaction than social media exposure alone. Lower SE was associated with both higher BMI and more frequent meals. This highlights potential pathways linking psychological wellbeing to eating patterns that warrant longitudinal investigation.

### 4.4. Null Moderation Result: Theoretical and Practical Implications

The central hypothesis—that self-esteem (SE) would moderate the social media use (SMU)–body satisfaction concerns (BSC) relationship—was not supported. This finding challenges the vulnerability–stress model assumption that individual characteristics such as SE invariably buffer or amplify environmental stressor effects (O’Reilly et al., 2018). Instead, the SMU–BSC association remained statistically equivalent across low, mean, and high SE levels. This suggests that social media exposure may be associated with comparable pressures regardless of self-worth levels in this population. Self-esteem has been identified as a critical protective buffer in other research. For instance, studies indicate that self-esteem partially mediates the relationship between problematic social media use and loneliness. The inclusion of psychological resources increases the explained variance of loneliness from 6% to 37% (Ingram & Luxton, 2005). The discrepancy between these findings and our results may reflect population differences, cultural contexts, or measurement variations. Importantly, the 95% CI [−0.005, 0.048] includes very small positive values that cannot be ruled out. Larger samples might yield different conclusions, though the effect would remain small (sr^2^ = 0.003).

Practically, interventions targeting SE alone may be insufficient to address social media-related body dissatisfaction. Multi-component approaches may be more effective. These should incorporate media literacy, critical consumption skills, and platform-specific content analysis.

Research utilizing PLS-SEM modeling among Generation Z users supports this necessity. Findings suggest that combining individual media literacy with cognitive control and supportive social environments is essential for navigating the information fragmentation of short video platforms (Yu et al., 2026). The comparable SMU–BSC associations across SE levels suggest that even students with relatively higher self-worth remain susceptible to social media pressures. This underscores the need for universal prevention strategies rather than risk-targeted approaches.

### 4.5. Limitations and Future Directions

Several methodological limitations constrain interpretation. First, the cross-sectional design precludes causal inference; all associations may reflect reverse association or third-variable confounding. Second, convenience sampling introduces selection bias, and self-report data entail social desirability and recall biases. Self-reported BMI likely underestimates actual values (Fayyaz et al., 2024). Third, while the questionnaires demonstrated strong internal consistency, formal psychometric validation of the Arabic translations was not conducted, undermining direct comparability with English-language literature. Fourth, the SMU questionnaire did not differentiate between platforms or content types, potentially obscuring platform-specific effects. Finally, the null moderation result (*p* = 0.113) should be interpreted cautiously given that the confidence interval includes near-zero positive values. Future research should: (1) employ longitudinal designs to establish temporal precedence; (2) conduct a formal psychometric validation study of the translated instruments using confirmatory factor analysis (CFA) to rigorously establish construct validity and evaluate measurement invariance across English and Arabic versions; (3) test whether social comparison or internalization of beauty ideals account for the SMU–BSC association; (4) examine alternative moderators such as media literacy or mindfulness; (5) use experience sampling to capture real-time social media use and momentary body image states; and (6) oversample male nursing students to assess gender-specific relationship patterns, as the current female majority (60.8%) may have obscured male-specific effects.

### 4.6. Study Implications

These findings inform intervention development and future research priorities. Female and second-year students showed slightly elevated SMU and BSC; targeted support during the transition to clinical training may help. However, the small effect sizes mean universal prevention approaches remain important. The significant correlations between SE and both SMU and BSC, combined with the non-significant moderation, suggest that SE may be a correlate rather than a protective buffer in this population. Interventions should therefore integrate SE enhancement with media literacy training rather than relying on either component alone. The associations with BMI and meal frequency highlight potential links between psychological wellbeing and eating patterns that merit longitudinal investigation. Campus health services should consider screening for body shape concerns and disordered eating patterns, particularly among students in high-stress academic years. Finally, the need for culturally adapted, validated Arabic versions of body image and social media measures remains pressing for future research in Middle Eastern populations.

## 5. Conclusions

This cross-sectional study of 324 Saudi student nurses found that female students reported marginally higher SMU and BSC than males, and second-year students showed the highest levels across all variables, though effect sizes were small to negligible. Higher SMU and greater BSC were each concurrently associated with lower SE, and BSC showed a stronger association with BMI than with SMU. Contrary to the vulnerability–stress hypothesis, SE did not moderate the SMU–BSC association, suggesting that the strength of this relationship did not vary across self-esteem levels. These findings underscore that demographic factors and the independent contributions of SMU and SE warrant consideration in addressing body shape concerns among nursing students. Interventions should combine self-esteem enhancement with media literacy training; strategies focused solely on self-esteem may not reduce social media-related body dissatisfaction. Future longitudinal research should examine temporal pathways and test whether alternative constructs—such as appearance comparison tendencies or media literacy—better explain individual differences in social media’s association with body image.

## Figures and Tables

**Table 1:** Exploratory Factor Analysis (EFA) Results.

Instrument	Factor Structure	EV	% Variance	α	RMSEA	CFI	SRMR
SMU (9 items)	Unidimensional	4.94	54.80%	0.88	0.048	0.972	0.041
BSQ-8A (8 items)	Unidimensional	4.93	61.60%	0.89	0.042	0.984	0.036
RSE (10 items)	General + method factor	5.19	51.90%	0.91	0.051	0.968	0.039

**Table 2:** Differences between the socio-demographics, social media, body shape concern, and self-esteem.

Variable	Category	N	M	SD	F	df1	df2	*p*	Effect Size
SMU	Gender				5.87	1	321	0.016	d = 0.07
	Female	197	20.3	8.71					
	Male	127	19.7	9.42					
	Year Level				3.21	3	320	0.023	η^2^ = 0.03
	First Year	91	20.2	9.04					
	Second Year	108	21	8.61					
	Third Year	73	18.8	8.84					
	Fourth Year	52	19.3	9.98					
BSC	Gender				4.55	1	321	0.034	d = 0.02
	Female	197	17.7	8.01					
	Male	127	17.5	8.86					
	Year Level				2.88	3	320	0.036	η^2^ = 0.03
Self-esteem	Gender				0	1	321	0.985	—
	Female	197	23.3	4.63					
	Male	127	23.3	4.72					
	Year Level				2.65	3	320	0.049	η^2^ = 0.02

**Table 3:** Correlation of SMU, Body shape concern, Self-Esteem, Meals, Time on social media, BMI, and Age.

Variables		SMU	BSC	SE
Meals per day				
rs		0.392 ***	0.373 ***	−0.240 ***
*p*		<0.001	<0.001	<0.001
Daily social media hours				
rs		0.275 ***	−0.105	−0.042
*p*		<0.001	0.058	0.446
BMI				
rs		0.154 **	0.506 ***	−0.189 ***
*p*		0.005	<0.001	<0.001
Age				
rs		0.012	0.173 **	−0.033
*p*		0.825	0.002	0.557
Variable Pair	rs	95% CI	*p*	Shared Variance (%)
SMU and BSC	0.393 ***	0.289, 0.491	<0.001	15.40%
SMU and SE	−0.334 ***	−0.435, −0.223	<0.001	11.20%
BSC and SE	−0.492 ***	−0.576, −0.400	<0.001	24.20%

Note. rs = Spearman’s rank correlation coefficient; CI = confidence interval; SMU = Social Media Use; BSC = Body Shape Concerns, SE = Self-Esteem, BMI = Body Mass Index ** *p* < 0.001. *** *p* < 0.01.

**Table 4:** Estimates of the Moderation Model.

Predictor	Estimate	SE	t	*p*	β	sr^2^	95% CI
SMU	0.4517	0.0634	7.13	<0.001	0.35	0.09	[0.327, 0.576]
SE	−0.6945	0.0989	−7.02	<0.001	−0.34	0.08	[−0.888, −0.501]
SMU × SE	0.0216	0.0136	1.59	0.113	0.03	0.003	[−0.005, 0.048]

Note. SE = standard error; t = test statistic; *p* = probability value; β = standardized regression coefficient; sr^2^ = semi-partial correlation squared (unique variance explained); CI = confidence interval; SMU = social media use; SE = self-esteem.

## Data Availability

The data presented in this study are available on request from the corresponding author.

## Appendix A

**Table A1:** A. Non-Significant Demographic Comparisons for Social Media Use, Body Shape Concern, and Self-Esteem (N = 324).

Variable	Category	N	M	SD	F	df1	df2	*p*	Effect Size
SMU	Household Income				1.57	4	316	0.182	—
	Internet at Home				1.37	1	322	0.243	—
	Area of Residence				0.13	1	322	0.719	—
BSC	Household Income				0.405	4	316	0.804	—
	Internet at Home				0.17	1	322	0.681	—
	Area of Residence				0.816	1	322	0.367	—
Self-esteem	Household Income				1.991	4	316	0.095	—
	Internet at Home				0.016	1	322	0.899	—
	Area of Residence				0.601	1	322	0.439	—

Note. SMU = Social Media Use; BSC = Body Shape Concern. Welch’s ANOVA used for all comparisons. All *p*-values > 0.05 indicate non-significant differences.
